# Otologic Manifestations and Progression in Patients with Wegener’s granulomatosis: A Survey in 55 Patients 

**Published:** 2017-11

**Authors:** Ali Safavi Naini, Jahangir Ghorbani, Sima Montazer Lotfe Elahi, Mohsen Beigomi

**Affiliations:** 1 *Chronic Respiratory Diseases Research Center, NRITLD, Masih Daneshvari Hospital, Shahid Beheshti University of Medical Sciences, Tehran, Iran.*; 2 *Chronic Respiratory Diseases Research Center, NRITLD, Masih Daneshvari Hospital, Shahid Beheshti University of Medical Sciences, Tehran, Iran.*; 3 *Orthopedic Surgeon, Akhtar Hospital, Shahid Beheshti University of Medical Sciences, Tehran, Iran* *. *

**Keywords:** Ear, Wegener’s granulomatosis, Vasculitis

## Abstract

**Introduction:**

Granulomatosis with polyangiitis (GPA; also known as Wegener’s granulomatosis) is a primary systemic vasculitis involving the ear, nose and throat system (ENT) and lower respiratory tract. Because of the lack of knowledge regarding the clinical findings of GPA due to the limited number of studies, the current study was designed to investigate the prevalence and nature of the otology manifestations in the disease course.

**Materials and Methods:**

In the current prospective study, patients with a diagnosis of GPA from 2012–2016 were included. A definitive diagnosis was made based on the history, physical examination (otomicroscopy, Rinne and Weber test), audiometry, tympanometry, cytoplasmic and perinuclear anti-neutrophil cytoplasmic antibody (C-ANCA and P-ANCA) investigations, and pathologic studies.

**Results:**

Twenty-seven male and 28 female patients aged 41.6±15.3 years were enrolled. Ear involvement was found in 20 patients (36.3%), and the most prevalent symptom was loss of hearing followed by otalgia and tinnitus. Tinnitus improved in none of the patients. The most prevalent sign was otitis serous followed by mastoiditis and external otitis. The most important audiometry finding was sensorineural hearing loss. Pathological studies using pulmonary samples were more useful for diagnosis.

**Conclusions:**

Precise clinical examination is crucial for the early diagnosis of GPA. Otological manifestations are common, especially loss of hearing and otitis serous, and can be the first sign of this disease. Early diagnosis can lead to better treatment of Wegener’s granulomatosis.

## Introduction

Granulomatosis in association with polyangiitis (GPA), known generally as Wegener’s granulomatosis, is a primary systemic vasculitis involving the ear, nose, and throat (ENT) system, as well as the upper and lower respiratory tract (1). Most patients (73–93%) suffer from ENT system involvement and seek medical intervention for these manifestations (2–4). Many of these patients are screened by an ENT surgeon at their first visit to the hospital (5, 6), and more than 80% have a rhinologic condition and 20–40% have otological problems (7). Diagnosis is made on the basis of clinical examination, serologic tests and histopathologic studies, and may last for a few weeks to several years (3,5,8,9). However, the delay in diagnosis is due to the fact that GPA is a rare disease with an annual incidence of 10 to 15 cases per million (9), and therefore may not be considered as a possible cause of a patient's illness. GPA usually occurs with nonspecific symptoms and is not recognized until it interferes with multiple organs. In recent years, the ability to measure anti-neutrophil cytoplasm auto-antibodies (ANCA) and the increased knowledge of physicians has led to a decreased risk of delayed diagnosis (10,11).Measurement of ANCA level is helpful in almost all patients with active and severe GPA, but is not widely used in people with isolated upper respiratory tract disease (12–14). The histopathologic diagnosis of the disease is also very challenging, and finding a full house of necrotizing granulomata with giant cells and neutrophil predominant vasculitis occurs rarely (15). In a limited percentage of patients with mild disease compared with severe cases, granuloma can be found in association with vasculitis (14). However, ANCA measurements and histopathologic studies are, in most cases, not sufficient for diagnosis, and it is necessary for patients to be carefully monitored in order to exclude any differential diagnosis.

Given that ear involvement is one the first manifestations of Wegener’s granulomatosis, and considering the rarity of the disease and the lack of similar studies in Iran, the current study was performed to examine each of the earliest manifestations in these patients. The purpose of this study was to investigate the usual and unusual manifestations of the ear in Wegener's patients.

## Materials and Methods

This study was performed in 55 patients referred to the ENT Clinic of Masih Daneshvari Hospital between 2012 and 2016 with a diagnosis of Wegener's granulomatosis. The diagnosis of GPA was confirmed based on the recommendations of the European Vasculitis Study Group regarding the division of ANCA vasculitis (16). Recording of biographies and clinical findings included detailed outcomes of ear examinations, as well as the results of Rinne and Weber tests and otomicroscopy. All patients were examined for vertigo, otalgia, tinnitus, sensory neural hearing loss, conductive hearing loss (CHL), tympanic membrane (TM) rupture, and external otitis, otitis media, mastoiditis, osteomyelitis, and facial paralysis. Patient files and paraclinical examinations were evaluated and compared with the patient's current condition. This study, was conducted with the aim of examining ear presentation in Wegener's granulomatosis patients. The results of the study will provide recommendations for ear care and warning signs, referral times for timely referrals and important demonstrations.

The study was a cross-sectional analytical study, and data were collected through a chart review using patient files. All findings were analyzed using SPSS 21 software. The prevalence of different presentations of the disease was investigated. Quantitative data are presented as mean ± standard deviation (SD). Subsequently, independent sample t-tests or Chi-square tests with a significance level of P<0.05 were utilized to analyze the data.

## Results

Of 78 patients referred with a GPA diagnosis over a period of 4 years, data from 23 patients were partially or fully missing. Of the remaining 55 patients with Wegener’s granulomatosis, five (9.1%) had independent ear manifestations, two had lung and ear manifestations (3,6%), and 48 patients had various other manifestations as the first involved organ, including lungs, kidneys, arteritis, trachea, lungs and kidneys, lungs and trachea, and lungs and sinuses.

In terms of the general presentation (not the initial manifestations), 20 patients had overall ear involvement, constituting 36.4% of all cases. Most of the 20 patients who were referred to the clinic with otologic manifestations complained of hearing loss or otalgia in 18 (90%) and 11 (55%) cases, respectively. Hearing loss remained unchanged in eight out of 18 patients during treatment, improved in two patients, and worsened in the other eight patients. Otalgia was stable in six patients. Furthermore, otalgia was exacerbated and improved in two and three patients, respectively. Other otological findings were tinnitus in eight patients (40%) and vertigo in four patients (20%). Tinnitus was the symptom most resistant to treatment (Table.1).

**Table 1:** Frequency of symptoms and progression in ear

**Symptoms**	**Frequency**	**Progression**		
		**No change**	**Worsening**	**Improvement**
Vertigo	4 (20%)	1	2	1
Hearing loss	18 (90%)	8	8	2
Tinnitus	8 (40%)	3	5	0
Otalgia	11 (55%)	6	2	3

Cholesteatoma was not observed in these patients, and the most common otological signs were serous otitis and mastoiditis in 13 (65%) and 8 (40%) patients, respectively. Among those patients who experienced a change during treatment, four had intensified signs and two had improved signs. Among the eight patients (40%) with mastoiditis, four remained unchanged, two recovered, and two had an exacerbation of signs. Other signs included external otitis, TM perforation, facial paresis, and osteomyelitis (Table.2).

**Table 2:** Frequency and progression of otological signs in patients

		**Progression**		
**Signs**	**Frequency**	**No change**	**Worsening**	**Improvement**
External otitis	6 (30%)	2	2	2
TM perforation	3 (15%)	1	1	1
Facial paresis	2 (10%)	1	0	1
Osteomyelitis	1 (5%)	1	0	0
Mastoiditis	8 (40%)	4	2	2
Serous otitis	13 (65%)	7	4	2
Cholesteatoma	0	-	-	-

Regarding audiometry and tympanometry, of 19 patients (95%) with sensorineural hearing loss (SNHL) symptoms (nine of which were bilateral), eight remained unchanged at the end of the course, five had symptom intensification, and six showed improvement.

Of 11 patients (55%) with concomitant CHL symptoms (four of which were bilateral), five were unchanged, four had symptom intensification, and two patients improved. Left and right tympanometry (B-type) were 10 (50%) and 7 (35%), respectively (Table.3). Ultimately, 83.6% of the patients responded to the treatment, but ear involvement was about 40%.

**Table 3:** Audiometry and tympanometry data

		**Progression**		
**Symptom**	**Frequency**	**No change**	**Worsening**	**Improvement**
SNHL	19 (95%) (9 bilateral)	8	5	6
CHL	11 (55%) (4 bilateral)	5	4	2
Rt. tympanometry (type B)	7 (35%)	4	2	1
Lt. tympanometry (type B)	10 (50%)	5	3	2

## Discussion

Wegener’s granulomatosis is a rare idiopathic condition resulting from changes in the small arteries, and has several clinical manifestations. Ear manifestations can be the first sign of the disease and Wegener’s granulomatosis should be considered in patients with resistant ear symptoms in order to achieve an early diagnosis. Early diagnosis can lead to better treatment and outcomes in patients. Otolaryngologist plays an important role in the early diagnosis and treatment of patients with ear manifestations.

In the current study, the response to treatment was investigated. In a study by Martinez Del Pero et al, in which 144 patients with Wegener disease were evaluated, ENT involvement was found in 87% of patients (17). Hearing loss and abnormal TM manifestation were also common in these patients. Wegener's disease is characterized by airway granulomatous vasculitis and glomerulonephritis. Despite progress in diagnosis and treatment, the cause is still unclear. Often head and neck involvement occurs prematurely or only with the appearance of the disease. The goal of the study conducted by Vega Braga in 2013 was to investigate signs and symptoms in the nasal area of ​​the valance of ganglia in Wegener's patients. Seventeen patients with Wegener's disease were examined. The mean age was 41.7 years and the mean duration of the disease was 12.9 years. Nine cases with hearing loss were found, of which five had bilateral dysfunctional hearing loss. In the nose, the nasal congestion and rhinorrhea were the most common manifestations. In the laryngopharynx, dyspnea in six cases and hoarseness in seven cases were the most prevalent findings. The authors concluded that the otolaryngologist plays an important role in the diagnosis, treatment, and follow up of these patients and that knowledge about the common manifestations leads to early diagnosis and better treatment (18).

Clinical symptoms of the disease were seen in all patients. C-ANCA was positive in all 20 patients and perinuclear anti-neutrophil cytoplasmic antibody (P-ANCA) assessment was positive in 14 (70%). From a pathological point of view, all results were positive, including one ear, two kidneys, three septum biopsies, and three trachea. The highest number of cases of positive biopsy (55%) was in the lung. An analytical study of six patients and review of the literature with Wegener's disease was performed in the Department of Rheumatology, at the Geral Hospital. The study included 49 patients. Systemic events were reported in 35 patients, and limited events in 14 patients. Acute clinical manifestations (symptoms less than 3 months before the diagnosis) were found in 41% of patients. Insidious presentation was seen in 59% of patients. The incidence of clinical manifestations in patients with systemic disease was 64% (upper airways, 36%; lung, 18%; kidney, 25%; eyes, 11%; and skin, 27%). In limited disease, the prevalence of manifestations was upper airway, 84%; lung, 15%; and eyes, 23%. As a result of the lack of specific symptoms, the diagnosis of patients with insidious presentation reduces mortality and morbidity in acute illness (19).

A study was conducted in seven cases of Wegener's disease with initial ear manifestations, who were referred to the ENT Department in Poznan from 2002 to 2008 with otitis media symptoms with effusion, facial paralysis, sensory neural hearing loss, hearing loss, or hearing loss associated with otorrhea. Elderly patients with a rash, which was later found to be generalized Wegener conflict, were reported to be in poor health (the first died after 2 months; the second died after 7 days; the third within 2 months of observation with pulmonary failure; and the fourth repoted kidney failure within 1 month). Patients with locally encountered disease were under their management for between 1 and 5 years.

In some Wegener patients, ear involvement is considered the first symptom. It is also important to consider Wegener's in patients with unusual inflammatory symptoms of ear. Ear infections are very dangerous in Wegener's disease, so the early diagnosis of the disease in the early stages is crucial. Localization or focal involvement in the ear may require less invasive treatment than multiple organ involvement (2). The cytoplasmic anti-neutrophil cytoplasmic antibody (C-ANCA) study is a useful method for diagnosing of Wegener's disease. Lung samples have a higher diagnostic value than other organs involved. Most patients with systemic involvement may respond to treatment, but in cases of ear involvement, the response to treatment was not satisfactory. Therefore, in patients with resistance to the treatment of ear, Wegener should be considered as one of the differential diagnoses.

Treatment for severe cases and remission maintenance by the rheumatologist consists of daily cyclophosphamide in doses of 2mg/kg per day orally for about 3 months (the dosage should be reduced in patients with renal insufficiency) combined with glucocorticoids given as prednisolone, 1mg/kg per day for the first month, followed by gradual tapering with discontinuation after 6–9 months. Then, after 3 months, 3–6 months of cyclophosphamide therapy is continued at a dose of 1mg/kg per day, then cyclophosphamide is stopped and switched to another agent for remission maintenance, such as azathioprine 2mg/kg per day. In the absence of toxicity, maintenance therapy is given for a minimum of 2 years after remission.

## Conclusion

Precise clinical examination is crucial for the early diagnosis of GPA, as it is shown that clinical findings are present in 100% of cases. Otological manifestations are common, especially loss of hearing and otitis serous, and can be the first sign of the disease. Early diagnosis can lead to better treatment of Wegener’s disease.
